# Supplementary material for: Diverse paths to broadly neutralizing antibody escape among HIV-1 strains
Source: Nat Microbiol. 2026 May 11;11(6):1573–84. doi: 10.1038/s41564-026-02347-x (PMC13236584; doi:10.1038/s41564-026-02347-x)
Supplement: Supplementary file 1 — Flow cytometry gating strategy. [file 41564_2026_2347_MOESM1_ESM.pdf]

nature microbiology

Supplementary information https://doi.org/10.1038/s41564-026-02347-x

Diverse paths to broadly neutralizing antibody escape among HIV-1 strains

In the format provided by the authors and unedited

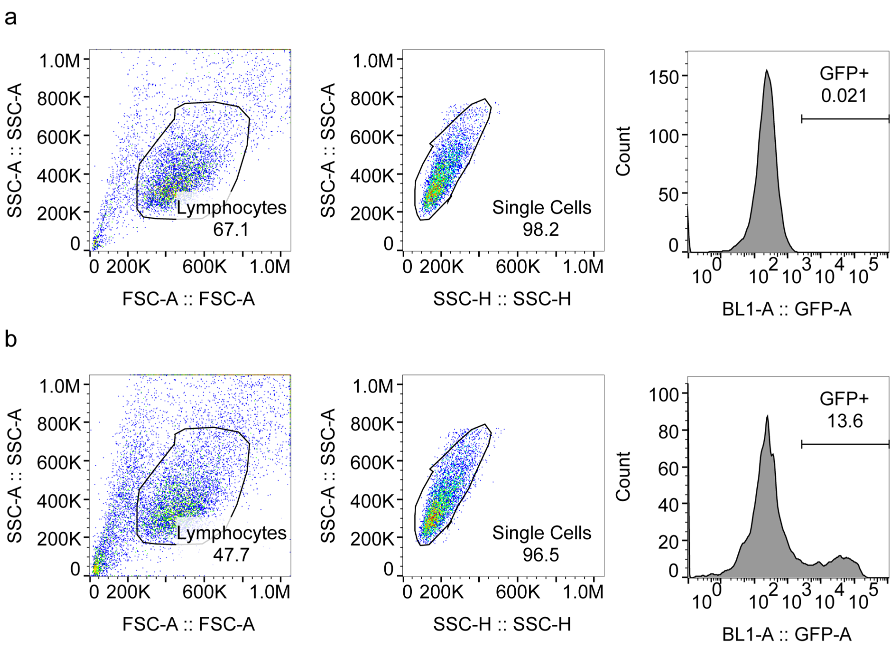

Flow cytometry gating strategy shown for identifying infected MT4-R5-GFP and MT4-R5-GFP-CD4high cells.
